# The E3 ubiquitin ligase TRIM31 attenuates NLRP3 inflammasome activation in *Helicobacter pylori*-associated gastritis by regulating ROS and autophagy

**DOI:** 10.1186/s12964-022-00954-9

**Published:** 2023-01-03

**Authors:** Qiao Yu, Huiying Shi, Zhen Ding, Zhe Wang, Hailing Yao, Rong Lin

**Affiliations:** Department of Gastroenterology, Union Hospital, Tongji Medical College, Huazhong University of Science and Technology, Wuhan, 430022 China

**Keywords:** *Helicobacter pylori*, TRIM31, Autophagy, ROS, NLRP3 inflammasome

## Abstract

**Background:**

The NLRP3 inflammasome activation is the molecular basis of *Helicobacter pylori* (*Hp*)-associated gastritis. Tripartite motif (TRIM) 31 is involved in diverse pathological events. However, whether TRIM31 plays a role in the activation of NLRP3 inflammasome in *Hp* infection is not clarified.

**Methods:**

A mouse model of chronic *Hp* infection was established, and the gastric tissues were subjected to the polymerase chain reaction, western blotting, histopathological analysis, and RNA sequencing. The mitochondrial membrane potential and ROS in the human gastric epithelium GES-1 cells with or without *Hp* infection were measured by flow cytometry. GES-1 cells with or without TRIM31 knockdown were transfected with mCherry-EGFP-LC3 adenovirus. After rapamycin and bafilomycin A1 stimulation, autophagy flux in the above primed GES-1 cells was assessed by laser confocal microscope. Lysosomal acidification and expression levels of cathepsin B and cathepsin D in GES-1 cells with *Hp* infection were measured.

**Results:**

NLRP3 inflammasome was activated in the gastric tissues of mice with chronic *Hp* infection in vivo and the GES-1 cells with *Hp* infection in vitro. TRIM31 was downregulated in *Hp* infection. TRIM31 negatively regulated the NLRP3 inflammasome activation. Enhanced ROS, impaired autophagy flux, and decreased expression of lysosomal cathepsin B and cathepsin D were observed in TRIM31-deficient GES-1 cells with *Hp* infection. In turn, inhibition of ROS led to the decreased expression of NLRP3 inflammasome.

**Conclusions:**

Together, our data identified that TRIM31 negatively regulated the activation of NLRP3 inflammasome in *Hp*-associated gastritis by affecting ROS and autophagy of gastric epithelial cells.

**Video abstract**

**Supplementary Information:**

The online version contains supplementary material available at 10.1186/s12964-022-00954-9.

## Background

*Helicobacter pylori* (*Hp*) colonizes the healthy human gastric epithelial cells of more than 50% of the world’s population [1]. *Hp* persistent infection is well known associated with complications containing chronic gastritis, ulcer diseases, and even gastric carcinoma [2, 3]. Chronic inflammation induced by *Hp* infection is regarded as the primary cause of the occurrence of gastric cancer [4–6]. Almost all of those *Hp* infections present chronic active gastritis with varying disease severity [7]. The initiation of pro-inflammatory signaling cascades within gastric epithelial cells during *Hp* infection remains unclear.

NLRP3 inflammasome is an oligomeric complex that consists of a NOD-like receptor NLRP3 sensor, a caspase-1 effector, and an ASC adaptor [8]. The activation of the NLRP3 inflammasome results in the caspase-1-mediated release of inflammatory mediators IL-1β and IL-18 [9]. As reported, the activation of the NLRP3 inflammasome and production of large amounts of the pro-inflammatory factor IL-1β are crucial factors for the occurrence and progression of *Hp*-associated gastritis and gastric cancer [10]. The precursor lesions, including *Hp*-associated gastritis, are important components of the risks of gastric cancer. Personalized treatment and prevention based on specific risks are the best strategies to reduce the mortality of gastric cancer [11]. Therefore, exploring the mechanism of NLRP3 inflammasome activation in *Hp*-associated gastritis is of great significance for further understanding the pathogenesis of *Hp*-associated gastritis, the inflammation-carcinoma chain, and the prevention of gastric cancer. However, the specific mechanism of the NLRP3 inflammasome activation in *Hp*-associated gastritis has not been fully elaborated.

The tripartite motif (TRIM) family proteins are composed of a RING-finger domain, 1–2 B-box domains, and a coiled-coil domain [12]. TRIM family controls a broad range of cellular processes [13]. As a member of the TRIM-containing proteins, TRIM31 is involved in diverse pathological events such as inflammatory diseases, protein quality control, autophagy, viral infection, and carcinoma development [14–17]. TRIM31 has been shown to attenuate the activation of the NLRP3 inflammasome by regulating ubiquitination of the relevant substrates [14]. However, in *Hp* infection, whether TRIM31 regulates the activation of NLRP3 inflammasome via novel signaling pathways is unknown.

Autophagy, which acts as a housekeeping role, is responsible for removing damaged organelles including mitochondria, peroxisome, and endoplasmic reticulum, as well as eliminating misfolded proteins and intracellular pathogens [18, 19]. It has been indicated that a wide range of upstream stimuli, such as ionic flux, lysosomal dysfunction, mitochondrial disruption, and release of mitochondrial reactive oxygen species (ROS) can trigger the NLRP3 inflammasome [20–25]. Autophagy has been demonstrated to negatively regulate the activation of the NLRP3 inflammasome by clearing endogenous trigger factors, such as impaired mitochondria that produce ROS [26, 27]. Owing to many upstream signals of NLRP3 inflammasome being interrelated or overlapping, as yet there is no highly unified opinion about NLRP3 inflammasome activation. Meanwhile, there is little information about a potent regulator of NLRP3 inflammasome in gastric epithelial cells of *Hp* infection subjects. Ra et al. [15] found that TRIM31 induces autophagy and promotes autolysosome formation in intestinal epithelial cells. However, whether TRIM31 is involved in the activation of the NLRP3 inflammasome by autophagy-related signaling pathways has never been clarified.

Therefore, in the current study, we intended to investigate the role and the specific mechanism of TRIM31 in NLRP3 inflammasome activation during *Hp* infection.

## Methods

### Animals

Four-weeks-old male BALB/c mice were obtained from Beijing Huafukang Bioscience Corporation (Beijing, China). All mice were maintained under standard specific pathogen-free conditions and were freely available for food and water. All experimental procedures were conducted according to the guidelines approved by the Animal Care and Use Committee and Animal Ethics Committee of Huazhong University of Science and Technology.

### Hp culture and supernatant collection

The *Hp* strain SS1 was kindly provided by the First Affiliated Hospital of Nanchang University. *Hp* was cultured using sterilized *Hp* liquid medium supplemented with 7% fetal bovine serum under a microaerobic condition at 37 °C and 200 rpm/min for 12–16 h. The *Hp* supernatants were collected after centrifuging at 4000 g for 15 min and filtered by 0.22 μM filters.

### Establishment of mouse models of chronic *Hp* infection

Mice were randomly allocated to 2 groups: the sham group and the chronic *Hp* infection group. In the chronic *Hp* infection group, mice were orally gavaged with *Hp* strain SS1 (3 × 10^9^ CFU/mL, 0.1 mL) once every other day for a total of 5 days (thrice in 5 days). In the sham group, mice were given *Hp* liquid culture medium following the same schedule. All mice were kept in standard laboratory conditions for 6 months.

### Cell culture conditions and stimulation

The GES-1 cells were cultured in Roswell Park Memorial Institute (RPMI 1640) medium containing 10% fetal bovine serum. In vitro, GES-1 cells were stimulated with the supernatants of *Hp* at a multiplicity of infection (MOI) of 100 for 12 h.

### Lentivirus transfection

Lentivirus of shRNA-TRIM31, TRIM31-overexpression, and each negative control was obtained from Obio Technology Corporation (Shanghai, China). The GES-1 cells were transfected with the above lentivirus using the Polyberene Transfection Reagent according to the manufacturer’s instructions. The cells were screened with puromycin for 7 days.

### Monomeric cherry (mCherry)–enhanced green fluorescent protein (EGFP)–LC3 transfection and confocal microscopy

mCherry–EGFP–LC3 adenoviruses (Hanbio, Shanghai, China) at MOI of 250 were transfected into wild-type and TRIM31-silenced GES-1 cells according to the manufacturer’s protocols. After rapamycin and bafilomycin A1 stimulation, cells were washed thrice with PBS and subjected to 4% paraformaldehyde fixation for 10 min. Next, the fixed cells on glass slides were treated with an anti-fade mounting solution (Antgene, Wuhan, China). The samples were immediately examined by fluorescence confocal microscopy (Nikon, Tochigi, Japan) and the NIS-Element Viewer software (Nikon, Tochigi, Japan).

### RNA extraction and real time-PCR (RT-PCR)

The total RNA was isolated from the gastric antrum of mice, or wild-type and TRIM31-deficient GES-1 cells that were either untreated or treated with *Hp* supernatant for 12 h using RNA-easy Isolation Reagent (Vazyme, Nanjing, China). The cDNA was synthesized using HiScript III-RT SuperMix kit (Vazyme, Nanjing, China). RT-PCR was conducted with SYBR qPCR Master Mix system (Vazyme, Nanjing, China). All samples were performed in a Roche LightCycler® 480 II PCR system. All data were normalized relative to levels of β-actin.

### Western blotting

Gastric tissue and GES-1 cells subjected to the respective treatments were obtained and homogenized in radioimmunoprecipitation assay lysis buffer supplemented with 1% Phenylmethanesulfonyl-fluoride protease inhibitor and phosphatase inhibitor on ice with intermittent vortex. Protein extracted from the supernatant was quantified with a bicinchoninic acid Kit (Vazyme, Nanjing, China). Denatured proteins were subjected to SDS-PAGE (EpiZyme, Shanghai, China) and electrotransferred to polyvinylidene fluoride membranes. Proteins on the membranes were incubated separately with appropriate antibodies overnight at 4 °C. Protein bands were visualized by ECL system (Vazyme, Nanjing, China).

### Immunohistochemistry

TRIM31 was detected by immunohistochemistry in paraffin-embedded gastric tissue slices. After deparaffinization, specimens were placed in a citric acid buffer and boiled for antigen repair for 2 min. 3% H_2_O_2_ was used to block the activity of endogenous peroxidase for 10 min, followed by goat serum blocking nonspecific sites for 30 min at room temperature. Specimens were incubated with monoclonal anti-TRIM31 primary antibody (1:800, Abclonal, USA) overnight at 4 °C. Then, the samples were washed with PBS and incubated with the second antibody, diaminobenzidine chromogenic agent, and hematoxylin staining in sequence according to the manufacturer’s protocols (Boster, Wuhan, China). Finally, photomicrographs were taken under a light microscope (OLYMPUS BX53).

### ELISA

IL-1β concentrations in cell culture supernatants were determined using Human IL-1β ELISA Kit (Hualianke, Wuhan, China) according to the manufacturer’s protocols.

### Mitochondrial membrane potential detection

Mitochondrial membrane potential-dependent MitoTracker Deep Red FM (YEASON, Shanghai, China) and membrane potential independent MitoTracker Green FM (YEASON, Shanghai, China) were used to measure mitochondrial membrane potential. Wild-type and TRIM31-deficient GES-1 cells were either untreated or primed with *Hp* supernatant for 12 h. Cells were cocultured with 50 nM MitoTracker Deep Red FM and 50 nM MitoTracker Green FM for 30 min. Then, cells were washed twice with PBS and treated with trypsin–EDTA. Single-cell suspension was examined by flow cytometry using a FACSCalibur (BD LSRFortessa X-20, BD Bioscience, USA). Stained mitochondria were analyzed according to the level of mean fluorescence intensity.

### Mitochondrial ROS detection

The presence of mitochondrial ROS was determined using MitoSOX Indicator (YEASON, Shanghai, China). In brief, GES-1 cells were firstly stimulated with *Hp* supernatants followed by washing twice with PBS. Secondly, cells were incubated with 4 µM MitoSOX for 10 min at 37 °C and washed twice. The level of mitochondrial ROS was measured by flow cytometry using a FACSCalibur (BD LSRFortessa X-20, BD Bioscience, USA).

### Lysotracker red staining

The culture medium of GES-1 cells treated with or without *Hp* supernatant for 12 h was removed. GES-1 cells were incubated with 75 nM LysoTracker Red DND-99 (YEASON, Shanghai, China) for 2 h. Next, the cells were washed twice and fixed with 4% paraformaldehyde. DAPI was used to stain the cell nucleus. The samples were viewed by fluorescence confocal microscopy (Nikon, Tochigi, Japan).

### Lysosomal pH detection

LysoSensor Yellow/Blue DND-160 dye was used to examine lysosomal pH according to instructions as previously [28].

### Cathepsin B activity

The activity of Cathepsin B was measured by the Cathepsin B Magic Red assay kit (Abcam, UK). After stimulation, the cells were washed with PBS and incubated with Cathepsin B Magic Red staining solution for 45 min in the dark. Then, the cells were washed and stained with Hoechst 33342 for 20 min. The samples were viewed under fluorescence confocal microscopy. Cathepsin B activity was assessed as fluorescence intensity.

### Statistical analysis

SPSS 21.0 statistical software and GraphPad Prism software were used for data analysis. Data from at least three independent experiments were expressed as mean ± SD. For difference comparisons between groups, a one-way ANOVA or independent-sample t-test was conducted. *P* < 0.05 was defined as statistically significant.

## Results

### NLRP3 inflammasome is activated in *Hp* infection

**Fig. 1:**
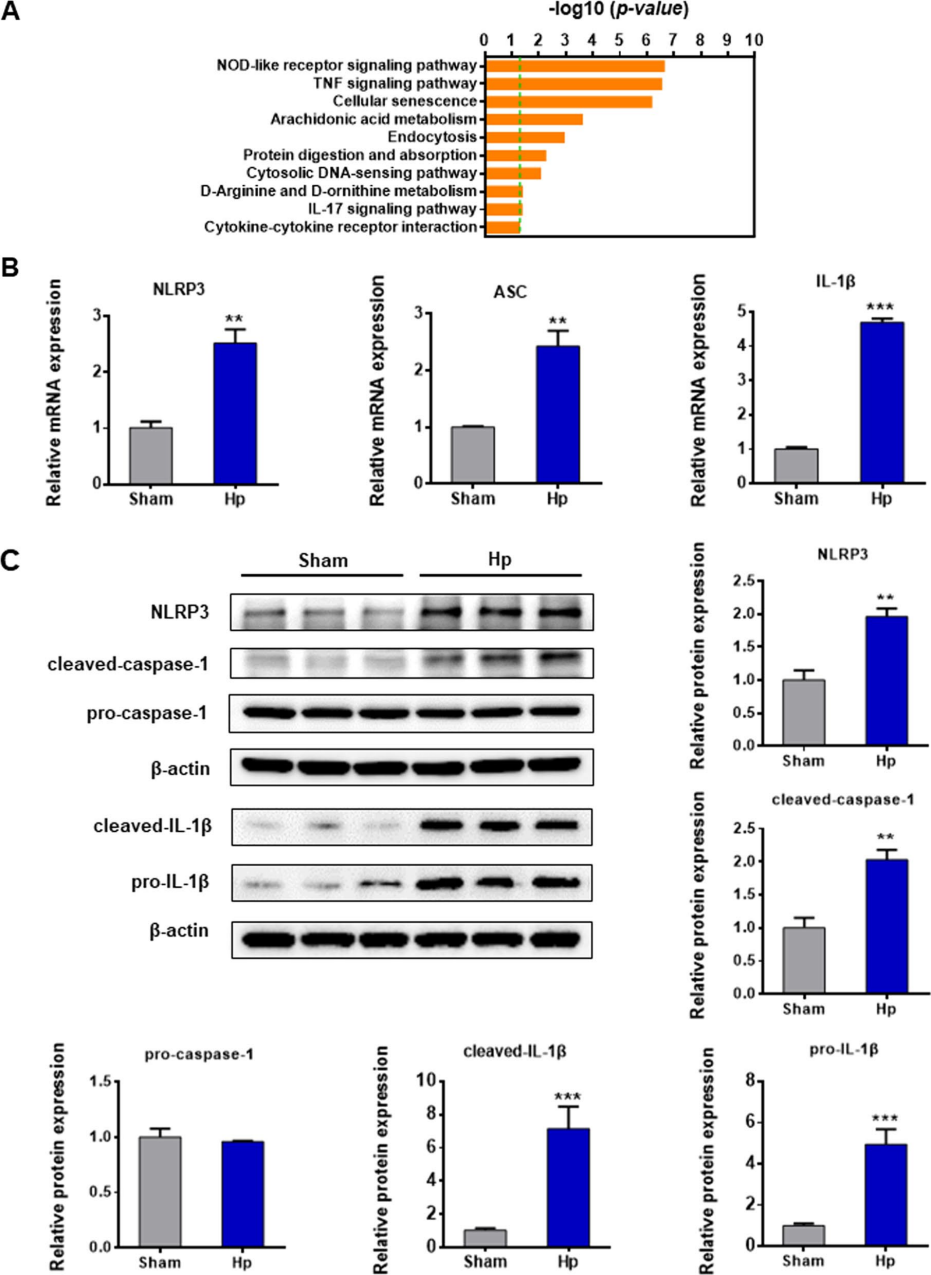
NLRP3 inflammasome was activated in gastritis caused by chronic *Hp* infection. **A** The differential pathway analysis of RNA-sequencing of gastric tissues of mice with chronic *Hp* infection (*Hp* group) and mice with the sham operation (sham group). **B** The mRNA expression of NLRP3, ASC, and IL-1β in gastric tissues of the *Hp* group and the sham group was measured by PCR. **C** The protein expression of NLRP3, cleaved caspase-1, pro-caspase-1, cleaved IL-1β, and pro-IL-1β in vivo was determined by western blotting. Results are expressed as means ± SD. **P < 0.01, ***P < 0.001

Firstly, we established a mouse model of gastritis caused by chronic *Hp* infection. HE staining and Giemsa dyeing assay confirmed that *Hp* colonizes the gastric mucosa of mice (Additional file 1: Fig. S1). The differential pathway analysis of RNA-sequencing data found that the NOD-like receptor signaling pathway showed significant statistical differences between the chronic *Hp* infection group and the sham operation control group (Fig. 1A). It was indicated that the NOD-like receptor signaling pathway is closely related to *Hp*-associated gastritis. As the critical member of the NOD-like receptor signaling family, the NLRP3 inflammasome plays a crucial role in the pathogenesis of *Hp*-associated gastritis.

In vivo, our results revealed that the mRNA expression of NLRP3, ASC, and IL-1β was increased in the chronic *Hp* infection group compared to the sham operation group (Fig. 1B). Western blot confirmed that the protein expression of NLRP3, cleaved caspase-1, and cleaved IL-1β was markedly increased in gastric tissue of *Hp* infection group. (Fig. 1C).

**Fig. 2:**
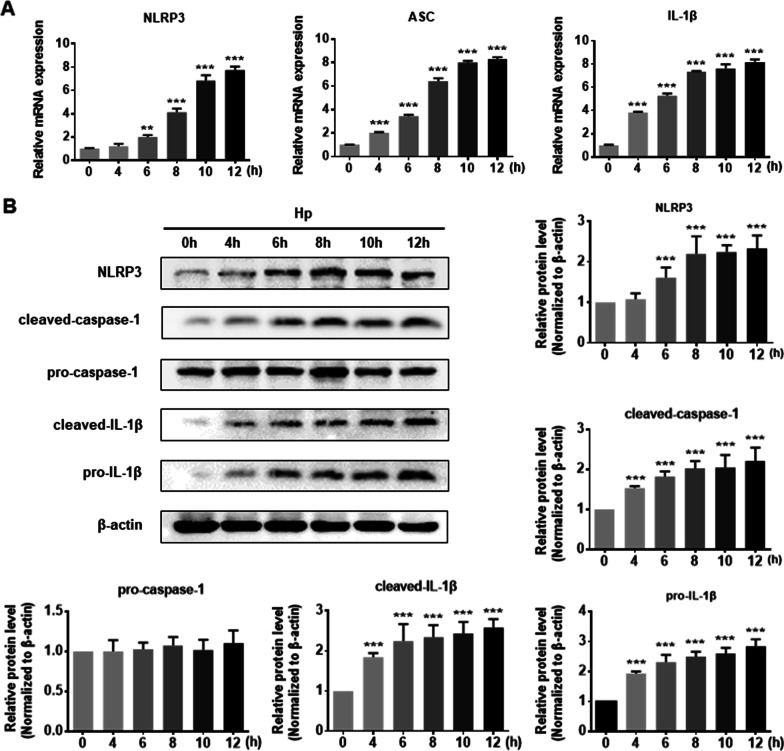
NLRP3 inflammasome was activated in GES-1 cells co-cultured with *Hp* supernatant. **A** The mRNA expression of NLRP3, ASC, and IL-1β in GES-1 cells treated with *Hp* supernatant for different duration times were measured by PCR. **B** The protein expression of NLRP3, cleaved caspase-1, pro-caspase-1, cleaved IL-1β, and pro-IL-1β in vitro was determined by western blotting. Results are expressed as means ± SD. **P < 0.01, ***P < 0.001

In vitro, the mRNA levels of NLRP3, ASC, and IL-1β in the GES-1 cells co-cultured with the *Hp* supernatant (*Hp* group) were higher than in the control group (Fig. 2A). In addition, the *Hp* infection enhanced the protein expression of NLRP3, cleaved caspase-1, and cleaved IL-1β in GES-1 cells in a time-dependent manner (Fig. 2B). Taken together, NLRP3 inflammasome is activated in *Hp* infection in vivo and in vitro.

### TRIM31 is downregulated in Hp-associated gastritis

**Fig. 3:**
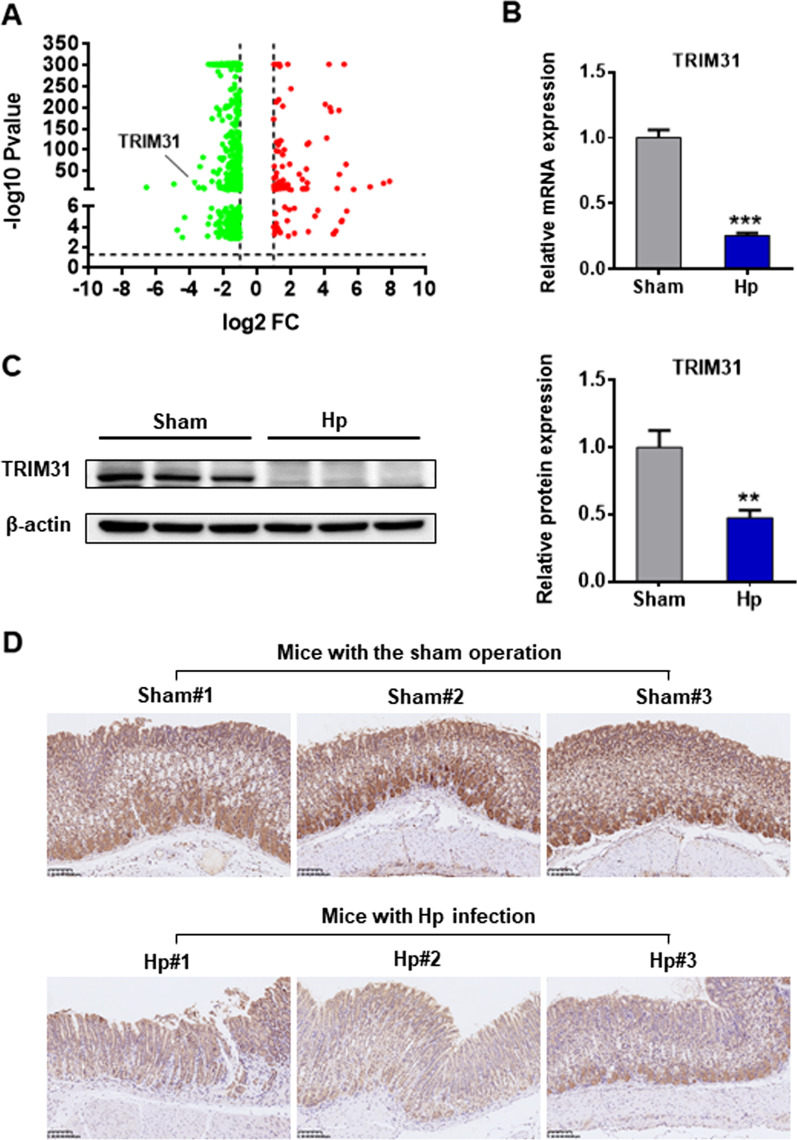
TRIM31 was downregulated in *Hp*-associated gastritis. **A** The differential gene analysis of RNA sequencing from gastric tissues of the chronic *Hp* infection group and the sham operation group. TRIM31 was significantly downregulated in *Hp* infection. **B** The mRNA expression of TRIM31 in gastric tissues of the *Hp* group and the sham group was measured by PCR. **C** The protein expression of TRIM31 in vivo was determined by western blotting. **D** The expression of TRIM31 in the *Hp* group and the sham group was assessed by the immunohistochemical method. Results are expressed as means ± SD. **P < 0.01, ***P < 0.001

NLRP3 inflammasome is activated in *Hp* infection, but the underlying mechanism remains unclear. To find the key genes involved in NLRP3 inflammasome activation, we analyzed the transcriptome data of the chronic *Hp* infection group and the sham group. The RNA-seq data showed that many genes were significantly suppressed by *Hp* infection, especially TRIM31 (Fig. 3A and Additional file 1: Fig. S2A). Confirming this, our data revealed that the mRNA and protein expression of TRIM31 was significantly downregulated in mice with *Hp*-associated gastritis (Fig. 3B–D).

### TRIM31 suppresses NLRP3 inflammasome activation in *Hp* infection

**Fig. 4:**
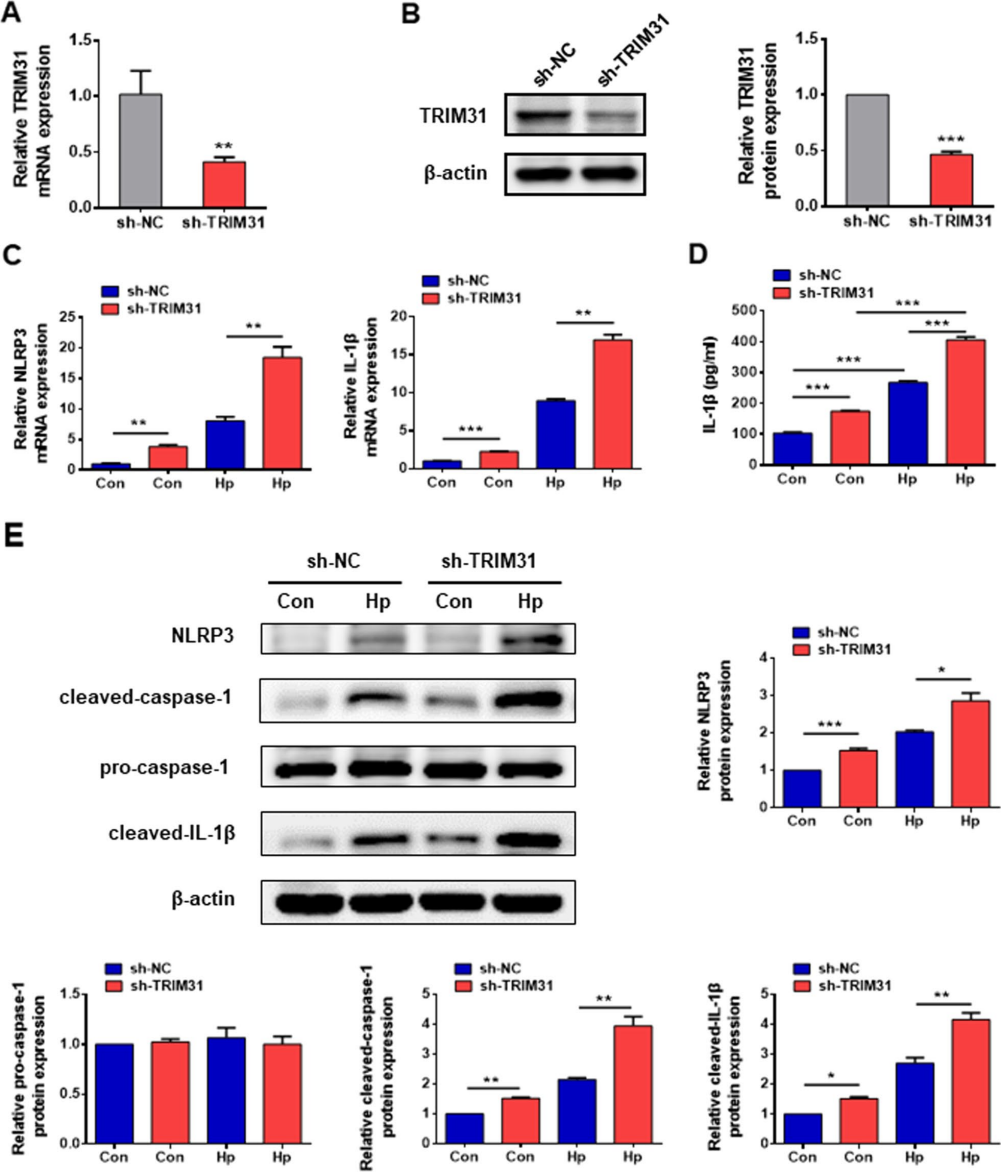
TRIM31 suppressed the activation of NLRP3 inflammasome and the production of IL-1β in GES-1 cells. The NC-shRNA and TRM31-shRNA lentivirus were transfected into the GES-1 cells. **A** The mRNA levels of TRIM31 in the sh-NC group and the sh-TRIM31 group were measured by PCR. **B** The protein expression of TRIM31 in the sh-NC group and the sh-TRIM31 group was determined by western blotting. **C** The mRNA levels of NLRP3 and IL-1β in the sh-NC group, the sh-TRIM31 group, the sh-NC + Hp group (sh-NC GES-1 cells treated with *Hp* supernatant for 12 h), and the sh-TRIM31 + Hp group (TRIM31-deficiency GES-1 cells treated with *Hp* supernatant for 12 h) were detected. **D** The level of IL-1β in the GES-1 cell culture supernatant was analyzed by ELISA. **E** The protein expression of NLRP3, pro-caspase-1, cleaved caspase-1, and cleaved IL-1β was measured by western blotting. Results are expressed as means ± SD. *P < 0.05, **P < 0.01, ***P < 0.001

A previous study has indicated that TRIM31 is the suppressor of NLRP3 inflammasome in sulfate-induced colitis [14]. We investigated whether TRIM31 influenced the activation of the NLRP3 inflammasome in *Hp* infection. To elucidate the role of TRIM31 in NLRP3 inflammasome activation, the effective TRM31-shRNA was used to inhibit endogenous TRIM31 expression in the GES-1 cells. The mRNA and protein levels of TRIM31 were significantly decreased, indicating that TRIM31-silenced GES-1 cells were successfully constructed (Fig. 4A, B). The IL-1β secretion and the mRNA expression of NLRP3 and IL-1β were significantly increased in TRIM31 silenced GES-1 cells primed by *Hp* supernatant (Fig. 4C, D). In addition, TRIM31 knockdown markedly enhanced the protein expression of NLRP3, cleaved caspase-1, and cleaved IL-1β in GES-1 cells (Fig. 4E). Similarly, TRIM31 overexpression greatly suppressed the protein expression of NLRP3, cleaved caspase-1, and cleaved IL-1β in GES-1 cells with *Hp* infection (Additional file 1: Fig. S2B). Together, these data confirmed that TRIM31 inhibited NLRP3 inflammasome activation and IL-1β secretion in *Hp* infection.

### Enhanced mitochondrial damage and ROS is observed in TRIM31-deficient GES-1 cells

**Fig. 5:**
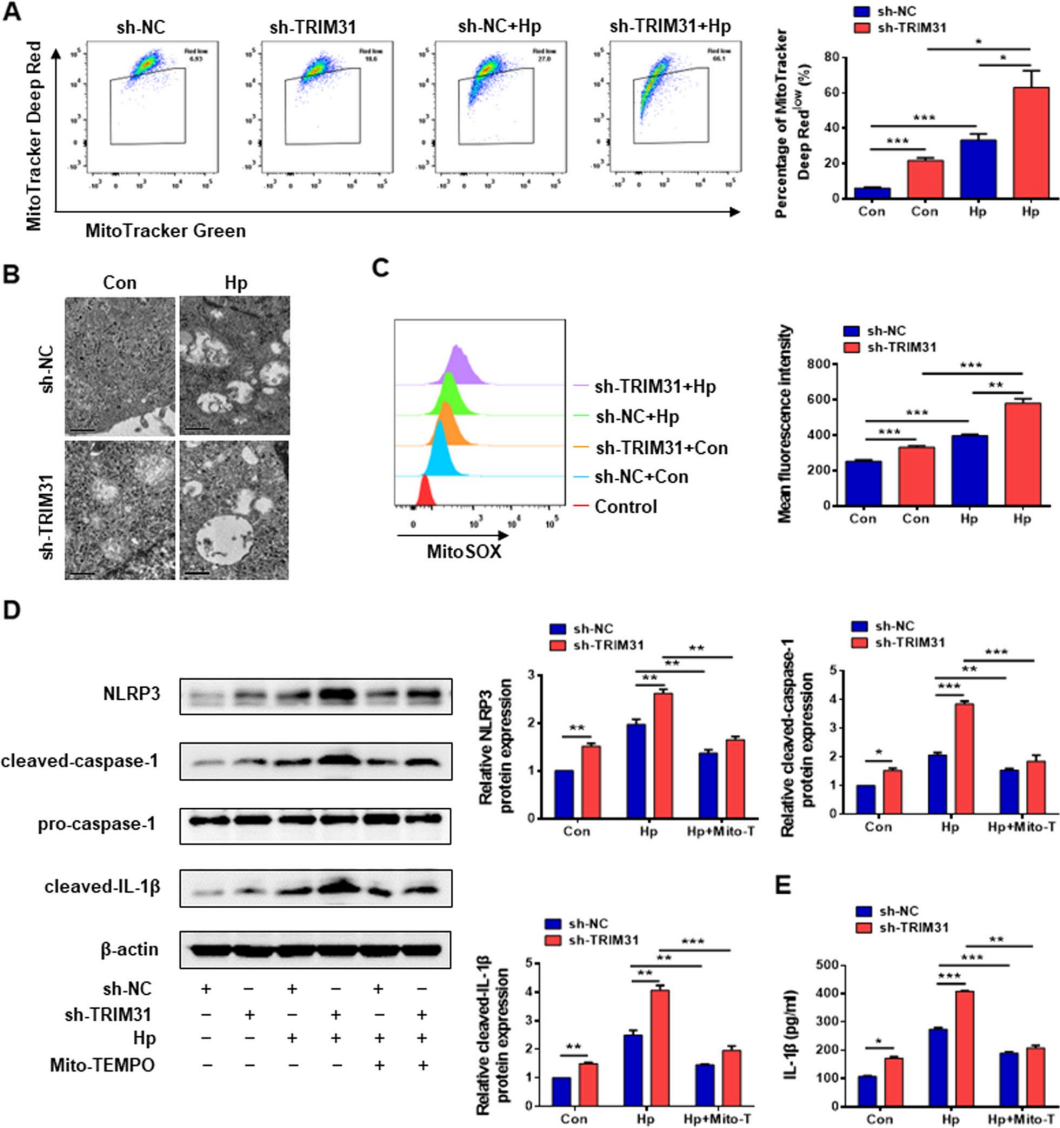
TRIM31 knockdown aggravated the mitochondrial damage and enhanced the production of mitochondrial ROS in GES-1 cells. **A** The mitochondrial membrane potential was measured using MitoTracker Deep Red and MitoTracker Green staining followed by flow cytometry. The percentage of MitoTracker Deep Red^low^ was used to assess the proportion of depolarized mitochondria. **B** The mitochondrial structure was observed by transmission electron microscopy. **C** MitoSOX was used to measure the level of mitochondrial ROS by flow cytometry. **D** Immunoblot analysis of NLRP3, pro-caspase-1, cleaved caspase-1, and cleaved IL-1β in different groups. Cells were primed with *Hp* supernatant alone or stimulated with *Hp* supernatant and Mito-TEMPO (500uM). **E** IL-1β concentration in the culture supernatant of sh-NC and TRIM31-silenced GES-1 cells was determined by ELISA. Results are expressed as means ± SD. *P < 0.05,**P < 0.01, ***P < 0.001

Then we continued to explore the specific mechanism of TRIM31 inhibiting NLRP3 inflammasome activation. The effect of TRIM31 on mitochondrial membrane potential was determined by flow cytometry. The proportion of depolarized mitochondria, indicated by a loss of MitoTracker Deep Red stain, was increased in the TRIM31-deficient GES-1 cells upon stimulation with *Hp* supernatant (Fig. 5A). Mitochondrial integrity in control and TRIM31-deficient GES-1 cells was assessed by transmission electron microscope. As shown in Fig. 5B, *Hp* infection resulted in mitochondrial swelling in sh-NC GES-1 cells, whereas the effect was strongly aggravated in TRIM31-silenced GES-1 cells, which were characterized by more pronounced mitochondrial swelling and the loss of cristae. The mitochondrial ROS level was assessed by MitoSOX using flow cytometry. Both *Hp* infection and TRIM31 knockdown significantly enhanced the production of mitochondrial ROS (Fig. 5C). Suppression of the mitochondrial ROS by Mito-TEMPO led to decreased protein expression of NLRP3, cleaved caspase-1, and cleaved IL-1β in the TRIM31-deficient GES-1 cells, as well as reduced IL-1β secretion (Fig. 5D, E). Together, these results suggested that TRIM31 deficiency enhanced the activation of NLRP3 inflammasome in GES-1 cells through the accumulation of damaged mitochondria and the production of mitochondrial ROS.

### TRIM31 knockdown contributes to impaired autophagy flux

**Fig. 6:**
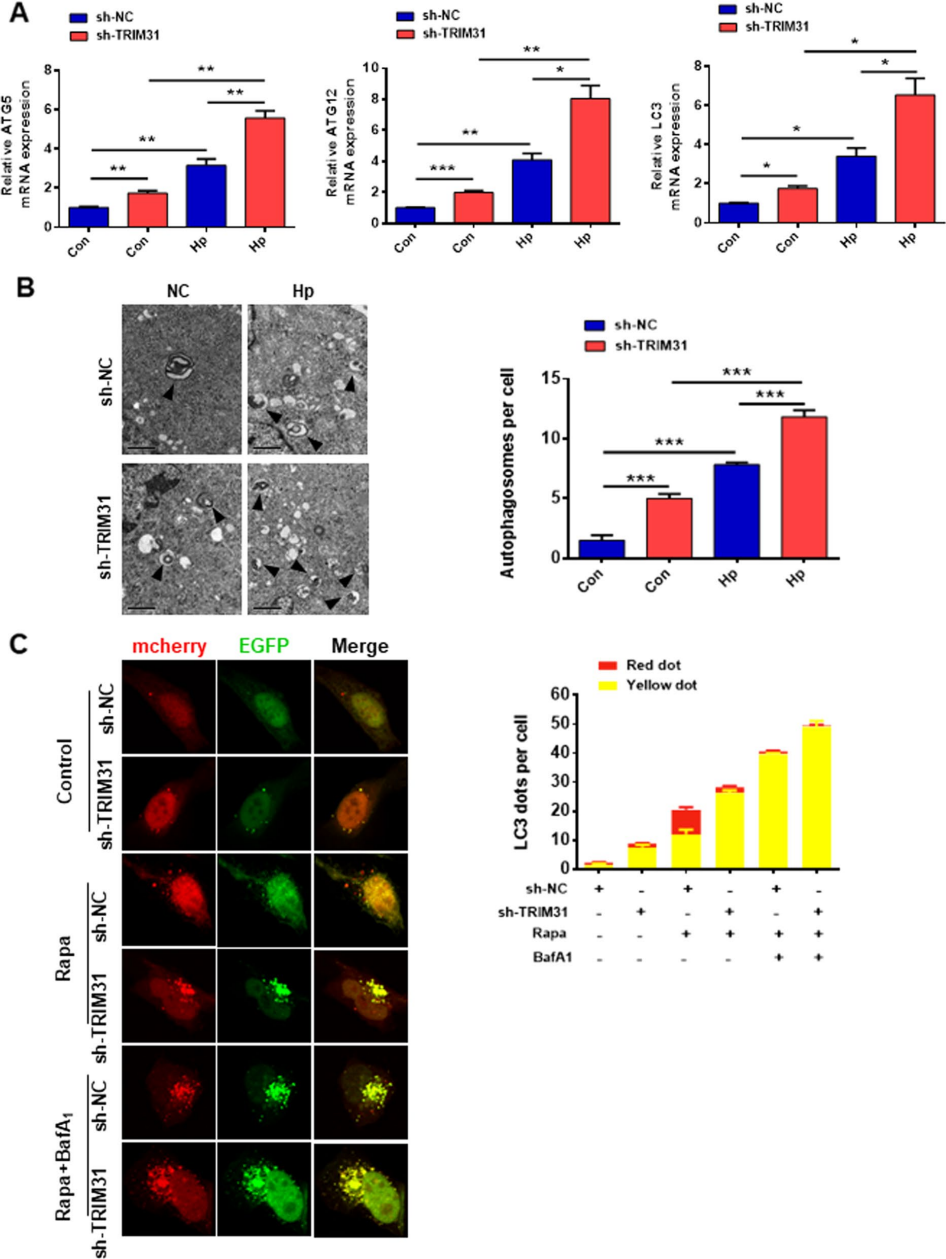
TRIM31 is required for autophagy. **A** The mRNA levels of autophagy-related genes ATG5, AT12, and LC3 were measured in sh-NC and TRIM31-deficient GES-1 cells treated with or without *Hp* supernatant for 12 h. **B** The formation of autophagosomes was analyzed by transmission electron microscopy. Representative images and the mean number of autophagosomes per cell (10 cells) were shown. **C** sh-NC and TRIM31-deficient GES-1 cells were transfected with mCherry-EGFP-LC3-expressing adenovirus. Cells were either unstimulated, treated with 100 ng/ml rapamycin (Rapa) alone, or 100 nM bafilomycin A1 (BafA1) in the presence of 100 ng/ml rapamycin for 8 h. The average number of autophagosome yellow dots (mCherry and EGFP colocalization) and autophagolysosome mCherry dots (EGFP was quenched in acidic lysosome) were determined manually (n = 10 cells). Results are expressed as means ± SD. *P < 0.05, **P < 0.01, ***P < 0.001

Autophagy plays a crucial role in the removal of damaged mitochondria. Therefore, we speculated that TRIM31 is involved in the autophagy process in GES-1 cells. The PCR analysis showed that the mRNA level of ATG5, ATG12, and LC3 were higher in the sh-TRIM31 + Hp group than in the sh-NC + Hp group (Fig. 6A). *Hp* infection resulted in an increase in the number of autophagosomes in TRIM31-deficient GES-1 cells compared to sh-NC GES-1 cells (Fig. 6B). Increased autophagy level or a defect in the autophagy completion was accounted for increased autophagosomes. To test this, an adenovirus plasmid encoding mCherry-EGFP-LC3 was transfected into sh-NC and TRIM31-deficient GES-1 cells. Under the steady-state, accumulated colocalized EGFP-mCherry puncta were observed in TRIM31-deficient GES-1 cells. After autophagy induction with rapamycin, the EGFP signal was quenched in the sh-NC GES-1 cells, indicating smooth autophagy flux or normal lysosomal delivery (which would result in degradation of the GFP). However, the majority of LC3 dots in TRIM31-deficient GES-1 cells were both mCherry and EGFP positive, indicating a defect of autophagy flux in TRIM31-deficient GES-1 cells (Fig. 6C). The addition of bafilomycin A1 blocked degradation in the late stage of autophagy in both sh-NC and TRIM31-deficient GES-1 cells (Fig. 6C). Collectively, these data indicated that TRIM31 knockdown led to impaired autophagy flux in GES-1 cells.

### TRIM31 is required for lysosomal maturation but not acidification

**Fig. 7:**
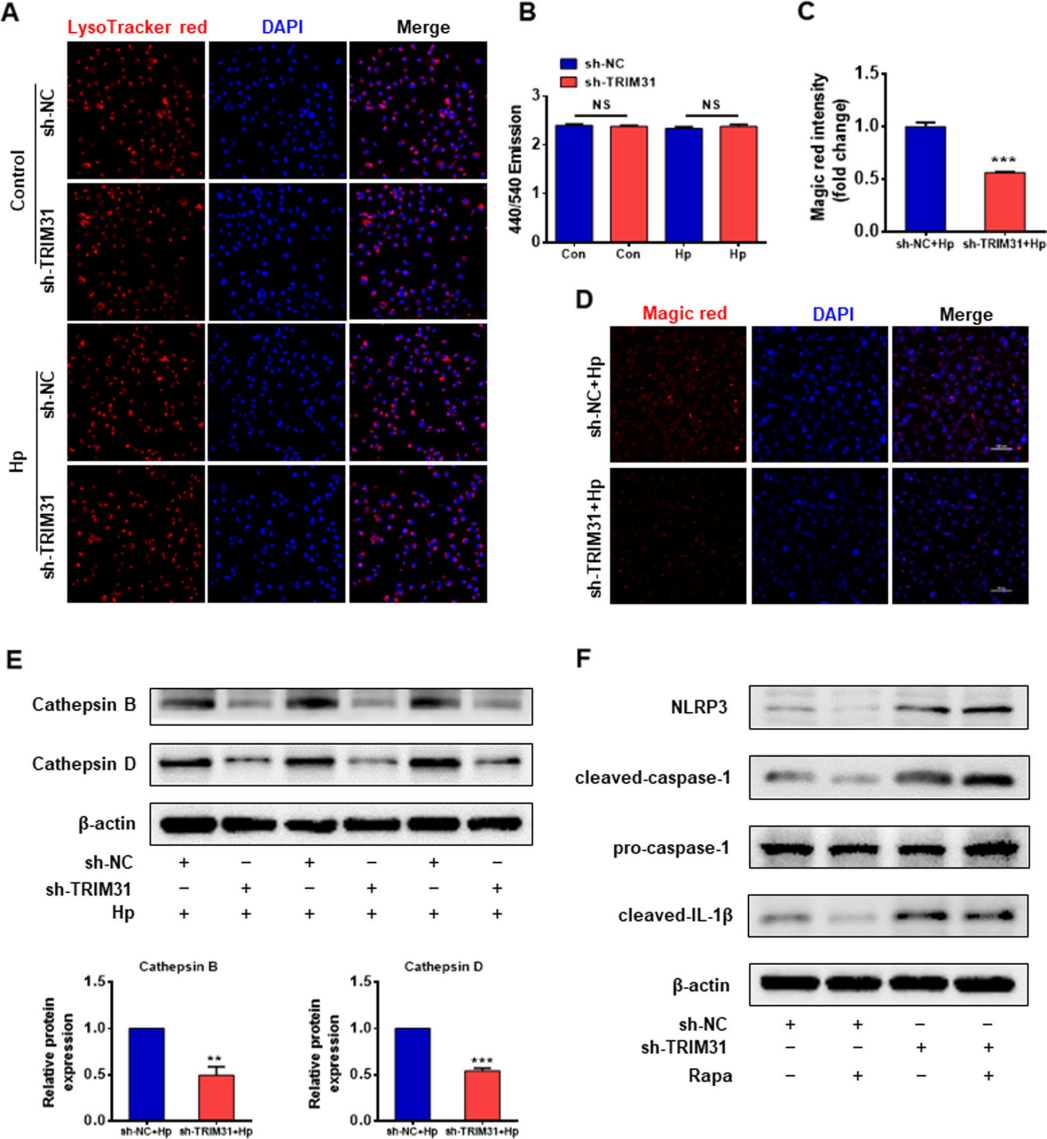
TRIM31 is required for lysosome maturation but not acidification. **A** LysoTracker Red staining was examined in sh-NC and TRIM31-deficient GES-1 cells that were either untreated or primed with *Hp* supernatant for 12 h. **B** To measure Lysosomal pH, sh-NC and TRIM31-deficient GES-1 cells primed with or without *Hp* supernatant for 12 h were treated with LysoSensor Yellow/Blue DND-160 dye and 440/540 emission was evaluated. **C**, **D** To assess Cathepsin B activity, live sh-NC and TRIM31-deficient GES-1 cells both treated with *Hp* supernatant were stained with the Cathepsin B Magic Red assay kit, and staining intensity was evaluated by a Confocal fluorescence microscope. **E** Immunoblot analysis of Cathepsin B and Cathepsin D in sh-NC and TRIM31-deficient GES-1 cells treated with *Hp* supernatant for 12 h. **F** Western blotting analysis of NLRP3, cleaved caspase-1, pro-caspase-1, and cleaved IL-1β in sh-NC and TRIM31-deficient GES-1 cells treated with or without rapamycin (100 ng/m) for 8 h. Results are expressed as means ± SD. NS, not significant, **P < 0.01, ***P < 0.001

Completion of autophagy is dependent on lysosomal function in the late stage of autophagy. We explored if TRIM31 influenced acidification and maturation of lysosomes in GES-1 cells with *Hp* infection. LysoTracker Red is a cell-permeable acidotropic probe that selectively labels vesicles with low internal pH. We evaluated the activity of the lysosomal compartment by using LysoTracker Red. As shown in Fig. 7A , a similar extent of accumulation of acidic vacuoles was observed in sh-NC and TRIM31-deficient GES-1 cells. LysoSensor Yellow/Blue DND-160 dye was used to assess lysosomal pH which is required for lysosomal function. There were no significant differences in lysosomal pH between sh-NC and TRIM31-deficient GES-1 cells (Fig. 7B).

In addition, we invest﻿igated whether the cathepsin activity was affected by TRIM31. Cathepsin B activity was measured by Magic Red staining. As indicated in Fig. 7C, D, the fluorescence intensity of Magic Red in the sh-TRIM31 + Hp group was significantly lower than in the sh-NC + Hp group, indicating that the proteolytic activity of cathepsin B was suppressed by TRIM31 knockdown. The protein expression of cathepsin B and cathepsin D was decreased in the sh-TRIM31 + Hp group compared to the sh-NC + Hp group (Fig. 7E). These results indicated that TRIM31 is required for lysosomal cathepsin activity but not acidification.

Finally, we explored whether TRIM31 inhibited the activation of the NLRP3 inflammasome by altering autophagy. The addition of rapamycin reduced the protein level of NLRP3, cleaved caspase-1, and cleaved IL-1β in sh-NC GES-1 cells, but to a lesser extent in TRIM31-deficient GES-1 cells (Fig. 7F). In conclusion, these data demonstrated that TRIM31 inhibited NLRP3 inflammasome activation by affecting autophagy in GES-1 cells with *Hp* infection.

## Discussion

The NLRP3 inflammasome activation and the IL-1β overexpression play crucial roles in the pathogenesis and development of *Hp*-associated gastritis [10, 29]. Damaged mitochondria, the production of ROS, and dysfunctional lysosome are the upstream events of NLRP3 inflammasome activation [30–32]. Autophagy is the negative regulator for NLRP3 inflammasome signaling [33]. In our study, we have determined that the activated NLRP3 inflammasome and excessive IL-1β secretion were observed in *Hp* infection*.* TRIM31 attenuates the NLRP3 inflammasome activation by suppressing the accumulation of damaged mitochondria and the production of ROS, as well as facilitating autophagy flux and maintenance of lysosomal function in *Hp* infection. Therefore, TRIM31 acts as a significant molecule to prevent inflammation for *Hp* infection.

NLRP3 inflammasome is closely associated with the progression of multiple diseases, such as *Hp* gastritis, gastric cancer, and inflammatory bowel disease [34–39]. Assembly of NLRP3 inflammasome resulted in the activation of caspase-1. Subsequently, active caspase-1 induces cleavage of pro-IL-1β and pro-IL-18, as well as gasdermin D, resulting in the release of biologically active IL-1β and IL-18 into the extracellular environment and the gasdermin D-induced the occurrence of pyroptosis [40–42]. The expression level of NLRP3 is variable in different cell lines or under different conditions. NLRP3 is expressed at very low levels in resting cells and is inadequate for initiating inflammasome activation under resting conditions [43]. When the NLRP3 inflammasome is activated, intracellular NLRP3 expression is significantly upregulated. Consistent with the previously reported study [39], our results showed that the expression level of NLRP3 was relatively low in GES-1 cells without *Hp* infection but significantly increased in GES-1 cells with *Hp* infection. Our data have demonstrated that the NLRP3 inflammasome was activated in *Hp-*associated gastritis in vivo and GES-1 cells with *Hp* infection in vitro. However, the mechanism of how NLRP3 inflammasome was activated in GES-1 cells during *Hp* infection was unknown.

TRIM31 has been reported to attenuate the activation of NLRP3 inflammasome in sulfate-induced colitis [14]. Our study showed that TRIM31 was downregulated in *Hp*-associated gastritis. TRIM31 suppressed the activation of NLRP3 inflammasome and the production of IL-1β in GES-1 cells with *Hp* infection.

Next, we explored the mechanism of how TRIM31 negatively regulated the NLRP3 inflammasome activation. TRIM31 is mainly located in the cytoplasm but some fraction was found involved in the mitochondria and lysosome [15, 16, 44]. Mitochondrial dysfunction, oxidative stress, NF-κB activation in gastric epithelial cells are typical events of *Hp* infection [45]. A range of upstream signals, including damaged mitochondria and ROS, were the activators of the NLRP3 inflammasome [24, 43, 46]. Our data indicated that both *Hp* infection and TRIM31 deficiency caused an increase in mitochondrial injury and the level of mitochondrial ROS and, but a decrease in mitochondrial membrane potential. Inhibition of mitochondrial ROS attenuates the NLRP3 inflammasome activation and IL-1β secretion in TRIM31-deficiency GES-1 cells, indicating that TRIM31 suppresses NLRP3 inflammasome activation by affecting mitochondrial dysfunction and ROS.

In vivo protein degradation involves two major systems: the autophagy-lysosome pathway and the ubiquitin–proteasome pathway [47, 48]. Autophagy is a protective process, involving removing aggregated proteins, impaired organelles (such as mitochondria), and destructing intracellular pathogens [49, 50]. Autophagy is a lysosomal degradation pathway that has an important influence on maintaining cell homeostasis, cell survival, and development [51]. The impaired autophagy can cause diseases with excessive activation of NLRP3 inflammasome and an active inflammatory state [26]. The molecules of the TRIM family involve extensive pathophysiology pathways such as autophagy [12, 52]. Our study demonstrated that TRIM31 deficiency led to autophagy flux blockade and decreased lysosomal degradation function. Activation of autophagy led to reduced NLRP3 inflammasome expression. Autophagy blockade resulted in the redundancy of dysfunctional mitochondria and ROS, which in turn triggers the NLRP3 inflammasome and initiates inflammatory reaction [24].

## Conclusions

**Fig. 8:**
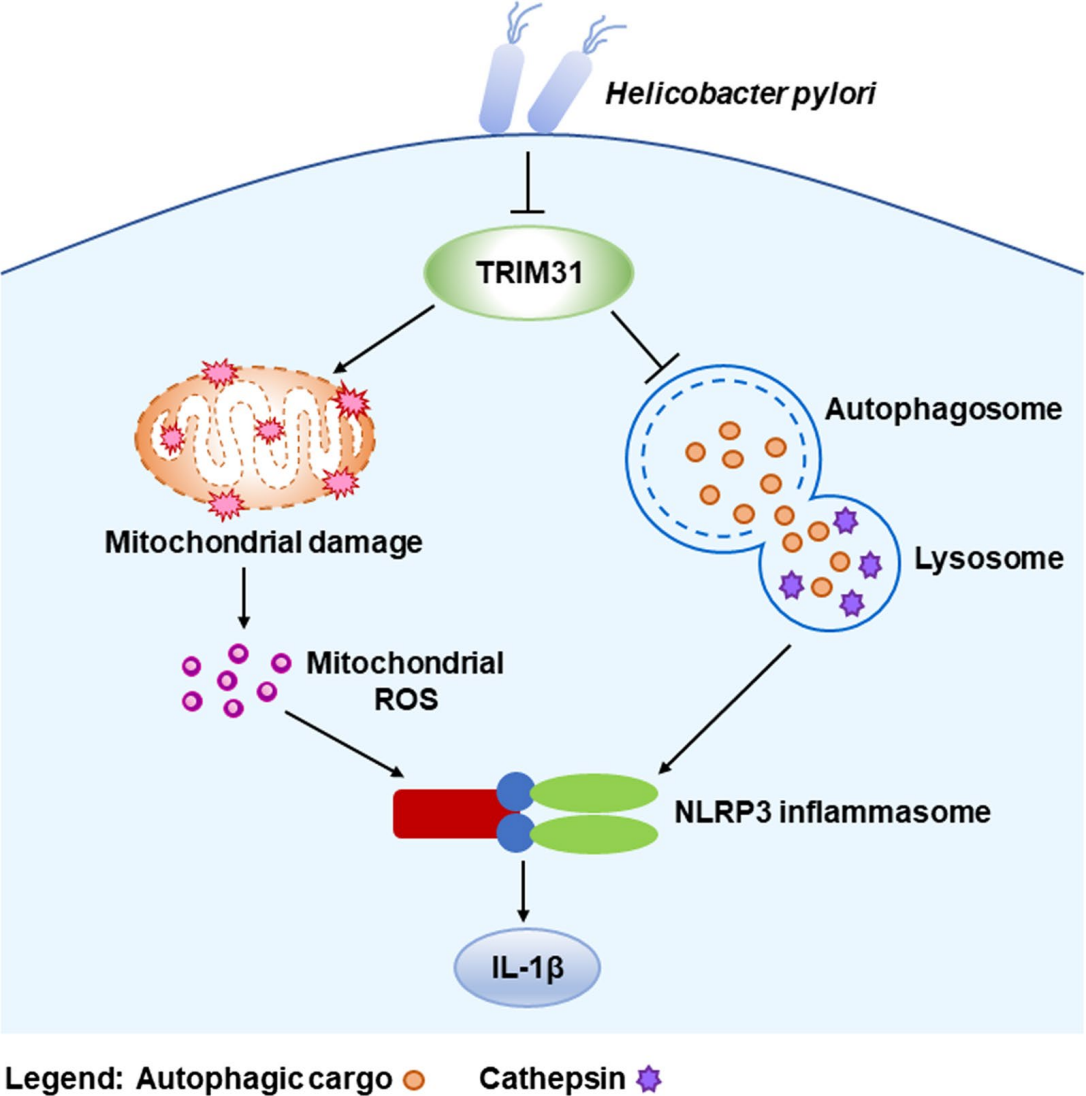
Schematic illustration depicting how TRIM31 acts upon NLRP3 inflammasome activation in *Hp*-associated gastritis by regulating ROS and autophagy. TRIM31 is downregulated in chronic *Hp*-associated gastritis. Increased ROS, impaired autophagy flux, and decreased levels of lysosomal cathepsin B and cathepsin D were observed in TRIM31-deficient GES-1 cells with *Hp* infection. TRIM31 suppressed the activation of the NLRP3 inflammasome by affecting ROS and autophagy in *Hp* infection

In summary, the current study points out that TRIM31 attenuates NLRP3 inflammasome activation in *Hp*-associated gastritis by affecting ROS and autophagy. Our results are summarized in a framework diagram (Fig. 8). *Hp* infection triggered the activation of NLRP3 inflammasome in GES-1 cells. TRIM31 was downregulated in *Hp* infection. TRIM31 knockdown enhanced the NLRP3 inflammasome activation and IL-1β secretion by aggravating mitochondrial damage, increasing mitochondrial ROS production, inducing obstructed autophagy flux, and reducing lysosomal degradation capacity. There are still some limitations in this study. Autophagy is a multi-step dynamic process involving many molecules. The key molecule in the autophagy process that interacts with TRIM31 remains unclear. Therefore, in future studies, we would like to explore which molecules interact with TRIM31 in the autophagy process in *Hp* infection. Our study describes a mechanism by which TRIM31 suppresses NLRP3 inflammasome activity in *Hp* infection. TRIM31 may act as a novel possible therapeutic target for *Hp*-associated gastritis.

## Supplementary Information

**Additional file 1**. **Figure S1**: The mouse model of chronic *Hp* infection and the mouse model of sham operation were established. Mice were orally gavaged with *Hp* strain SS1 or *Hp* liquid culture medium and maintained in the standard laboratory for 6 months. Representative photomicrographs of HE staining and Giemsa staining of the gastric tissue sections of the chronic *Hp* infection group and the sham operation group were shown. **Figure S2**: TRIM31 overexpression attenuated the activation of NLRP3 inflammasome in *Hp* infection. (A) Bar plot showing fold changes of genes that are significantly suppressed by *Hp* infection in the RNA-seq data. (B) The western blot assay of the expression of TRIM31, NLRP3, cleaved-caspase-1, pro-caspase-1, cleaved-IL-1β, and pro-IL-1β in TRIM31-overexpressing GES-1 cells with *Hp* infection or control GES-1 cells with *Hp* infection.

## Data Availability

All data analyzed during this study are included in this article.

## Abbreviations

*Hp*: *Helicobacter pylori*
ROS: Reactive oxygen species
PCR: Polymerase chain reaction
PBS: Phosphate buffered saline
MOI: Multiplicity of infection
