# Supplementary material for: The E3 ubiquitin ligase TRIM31 attenuates NLRP3 inflammasome activation in Helicobacter pylori-associated gastritis by regulating ROS and autophagy
Source: Cell Commun Signal. 2023 Jan 3;21:1. doi: 10.1186/s12964-022-00954-9 (PMC9809066; doi:10.1186/s12964-022-00954-9)
Supplement: Supplementary file 2 — Additional file 1. Figure S1: The mouse model of chronic Hp infection and the mouse model of sham operation were established. Mice were orally gavaged with Hp strain SS1 or Hp liquid culture medium and maintained in the standard laboratory for 6 months. Representative photomicrographs of HE staining and Giemsa staining of the gastric tissue sections of the chronic Hp infection group and the sham operation group were shown. Figure S2: TRIM31 overexpression attenuated the activation of NLRP3 inflammasome in Hp infection. (A) Bar plot showing fold changes of genes that are significantly suppressed by Hp infection in the RNA-seq data. (B) The western blot assay of the expression of TRIM31, NLRP3, cleaved-caspase-1, pro-caspase-1, cleaved-IL-1β, and pro-IL-1β in TRIM31-overexpressing GES-1 cells with Hp infection or control GES-1 cells with Hp infection. [file 12964_2022_954_MOESM2_ESM.docx]

**
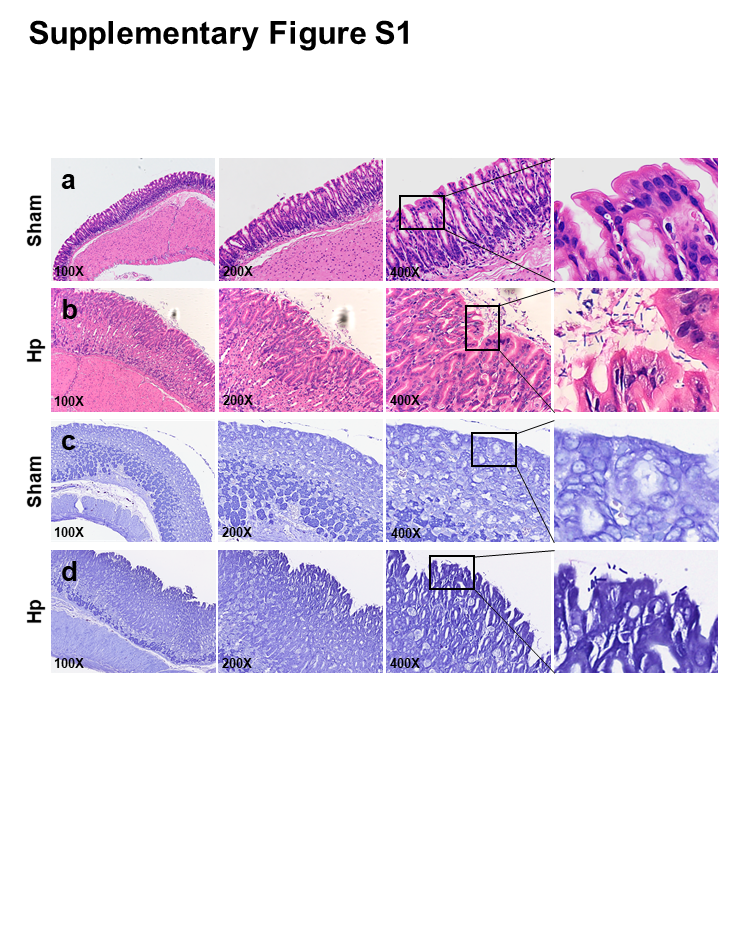
**

**Figure S1.** The mouse model of chronic *Hp* infection and the mouse model of sham operation were established. Mice were orally gavaged with Hp-SS1 strain or PBS and maintained in the standard laboratory for 6 months. Representative photomicrographs of HE staining and Giemsa staining of the gastric tissue sections of the chronic *Hp* infection group and the sham operation group were shown.

**
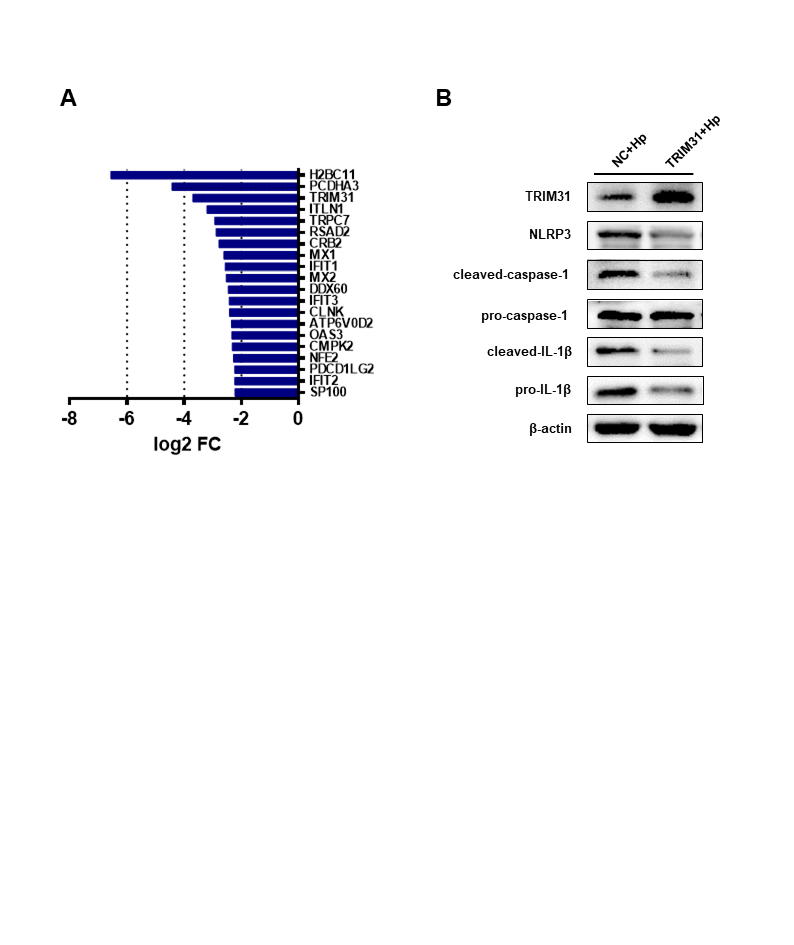
**

**Figure S2.** TRIM31 overexpression attenuated the activation of NLRP3 inflammasome in *Hp* infection. **(A)** Bar plot showing fold changes of genes that are significantly suppressed by *Hp* infection in the RNA-seq data. **(B)** The western blot assay of the expression of TRIM31, NLRP3, cleaved-caspase-1, pro-caspase-1, cleaved-IL-1β, and pro-IL-1β in TRIM31-overexpressing GES-1 cells with *Hp* infection or control GES-1 cells with *Hp* infection.
